# Benchmarking publicly accessible large language models for English-language patient-facing acute pancreatitis information: a cross-sectional study of quality, transparency, and readability

**DOI:** 10.3389/fpubh.2026.1880639

**Published:** 2026-08-17

**Authors:** Biao Jiang, Hongxi Sun, Linlin Chen

**Affiliations:** 1 Ward I, Department of Gastroenterology, Suining Central Hospital, Suining, Sichuan, China; 2 Stomatology Center, Suining Central Hospital, Suining, Sichuan, China

**Keywords:** acute pancreatitis, artificial intelligence, health information quality, large language models, patient education, readability, transparency

## Abstract

**Background:**

Patients increasingly rely on large language models (LLMs) for health information, yet their suitability for decision-critical conditions such as acute pancreatitis remains unclear. Given that acute pancreatitis requires timely symptom recognition, severity assessment, treatment decision-making, recurrence prevention, and follow-up management, LLM-generated information should demonstrate reliability, transparency, and readability.

**Objectives:**

To evaluate the informational quality, visible transparency-related features, and readability of English-language responses generated by five publicly accessible LLMs to standardized patient-facing questions on acute pancreatitis.

**Methods:**

This cross-sectional benchmark study developed 24 English-language, single-intent questions on acute pancreatitis across six clinical domains using public search intents and guideline-derived decision-critical content. The analysis focused exclusively on English-language patient-facing responses. Each question was submitted once to GPT-5.4 Thinking, DeepSeek-V3.2, Gemini 3.1 Pro, Grok 4.3, and Qwen3.6-Max-Preview, generating 120 responses. Anonymized responses were independently evaluated by two blinded gastroenterologists using DISCERN, Ensuring Quality Information for Patients (EQIP), Global Quality Scale (GQS), and Journal of the American Medical Association (JAMA) benchmark criteria. Readability was assessed using six established formulas. A structured response-level safety analysis was added to evaluate factual inaccuracies, clinical hallucinations, and clinical safety-risk severity. Between-model differences were analyzed with Friedman tests followed by *post hoc* paired Wilcoxon signed-rank tests with Holm correction.

**Results:**

Significant between-model differences were observed across all quality, transparency-related, and readability outcomes. Grok 4.3 achieved the highest mean DISCERN, GQS, EQIP, and JAMA scores, reflecting the strongest informational quality profile according to the predefined quality instruments, although visible transparency cues remained limited across all models. In the added safety analysis, factual inaccuracies and clinical hallucinations were each identified in 16 of 120 responses (13.3%), whereas clinical safety-risk signals were identified in 7 responses (5.8%), all of which were adjudicated as score 1 (low risk) under the predefined clinician-rated rubric; no moderate- or high-risk clinical safety event was adjudicated. DeepSeek-V3.2 demonstrated the most favorable readability profile, with the highest Flesch Reading Ease score (44.42 ± 12.27), which nevertheless remained substantially below the recommended threshold of ≥ 80. None of the 120 responses satisfied all six predefined readability targets. All model-level readability distributions differed significantly from recommended thresholds in the direction of poorer readability.

**Conclusion:**

Publicly accessible LLMs generated English-language responses on acute pancreatitis with variable informational quality, limited visible transparency cues, and consistently inadequate readability under standardized default public-interface conditions. Because the primary analysis used a single-generation cross-sectional design, model rankings should be interpreted as performance snapshots rather than definitive or temporally stable hierarchies. Higher quality scores did not necessarily establish factual accuracy, clinical safety, patient-education-level readability, or stronger response-level transparency. Current public-interface LLM outputs may serve as clinician-reviewed drafts for patient education but should not function as standalone patient resources. Future AI-based health information systems should strengthen clinical completeness, plain-language communication, visible evidence support, actionability, and clinician-supervised safeguards.

## Introduction

Acute pancreatitis is a common gastrointestinal emergency with a clinical spectrum ranging from self-limited pancreatic inflammation to persistent organ failure, infected necrosis, recurrent disease, and long-term complications (1, 2). Contemporary guidelines emphasize that safe management requires early diagnosis and severity stratification, selective imaging, individualized fluid resuscitation, early enteral nutrition when clinically appropriate, avoidance of routine antibiotics in the absence of infection, timely biliary intervention in selected patients, and recurrence prevention after discharge (3, 4). These recommendations are clinically nuanced and frequently depend on conditional reasoning rather than simple binary decisions. Consequently, patient education on acute pancreatitis should address not only the nature of the disease, but also when symptoms warrant urgent evaluation, why certain tests or treatments may not be immediately necessary, under what circumstances procedures such as endoscopic retrograde cholangiopancreatography are indicated, how oral feeding is resumed, and why underlying biliary or metabolic etiologies require continued management despite apparent recovery. Recent patient-centered studies further demonstrate that individuals with acute pancreatitis have substantial health-literacy needs spanning diagnosis, treatment, discharge, recovery, and recurrence prevention, highlighting the importance of clear, actionable patient education (5).

Large language models (LLMs) are increasingly used as conversational sources of health information and may provide scalable support for patient education (6–8). Previous studies and reviews indicate that LLMs can generate coherent, detailed, and occasionally empathetic responses to patient inquiries, while also highlighting persistent concerns regarding accuracy, hallucination, bias, reproducibility, readability, source attribution, and clinical oversight (7, 9, 10). These concerns are particularly relevant in acute pancreatitis, where patients may rely on online or AI-generated information to interpret warning symptoms, determine whether urgent medical attention is needed, understand imaging and laboratory findings, evaluate treatment decisions, and plan post-discharge follow-up. In this context, fluent and authoritative-sounding responses may still be difficult to comprehend, insufficiently transparent, or lacking appropriate safety safeguards.

Evaluating LLM-generated patient information requires more than determining whether a response appears medically plausible. Informational quality, visible transparency, and readability constitute related yet distinct dimensions of patient-facing utility. A response may be clinically comprehensive and detailed while still exceeding recommended reading levels, whereas a simpler response may improve readability at the expense of important decision-related information. Transparency is similarly critical because patients often evaluate the response itself rather than separate platform documentation. The Journal of the American Medical Association (JAMA) benchmark was originally developed to assess accountability in online health information through authorship, attribution, disclosure, and currency (11). Although LLM-generated responses differ from conventional health websites, the presence or absence of visible cues related to sourcing, disclosure, and currency remains relevant when assessing unprompted outputs. Concurrently, recent studies suggest that AI-generated health information frequently exceeds recommended patient-education reading levels, indicating that fluency and apparent completeness do not necessarily translate into accessible patient communication (12–15).

Despite increasing interest in LLM-assisted patient education, disease-specific evidence in acute pancreatitis remains limited. This gap is clinically significant because acute pancreatitis combines common public information needs with high-risk, decision-critical topics, including recognition of clinical deterioration, early severity assessment, computed tomography utilization, antibiotic decision-making, biliary intervention, refeeding strategies, recurrence prevention, and urgent post-discharge reassessment. A structured benchmark integrating both lay information-seeking intents and guideline-derived clinical content may therefore better reflect the questions patients are likely to ask and the responses that warrant careful evaluation. Whether models producing higher-quality or more clinically complete responses also provide patient-education-level readability and visible transparency-related cues remains unclear. Accordingly, this cross-sectional benchmark study evaluated and compared the quality, visible transparency-related features, and readability of English-language responses generated by five publicly accessible LLMs to standardized patient-facing questions on acute pancreatitis. These English-language outputs were assessed using DISCERN, Ensuring Quality Information for Patients (EQIP), Global Quality Scale (GQS), JAMA benchmark criteria, and six established English-language readability formulas.

## Methods

### Study design

This cross-sectional benchmark study evaluated the quality, transparency-related features, and readability of English-language responses generated by five publicly accessible LLMs to a standardized set of patient-facing acute pancreatitis questions. The study was designed to simulate an English-language public-user, zero-shot, single-turn information-seeking scenario under default web-interface conditions, in which a patient submits a question once and receives one immediate response. Accordingly, the primary analysis used one response per model-prompt pair to approximate this default public-user scenario rather than to estimate the full distribution of possible outputs across repeated generations.

### Question set development

The benchmark question set was developed using a prespecified domain-balanced and source-balanced framework rather than frequency-weighted sampling of public queries. This approach was intended to ensure representation of lower-frequency but clinically high-risk tasks, including recognition of clinical deterioration, triage, and time-sensitive management decisions, which might otherwise be underrepresented. Six clinical domains were predefined: (1) symptoms and thresholds for seeking care, (2) causes and recurrence, (3) diagnosis and severity assessment, (4) early treatment, (5) nutrition and inpatient management, and (6) complications and post-discharge management.

Within each domain, one final question was derived from each of four source categories—Google Trends, Baidu Zhidao, Chinese guidelines, and international guidelines—yielding a 24-item benchmark set. The Chinese guideline anchor was based on the 2021 Chinese guideline identified through PubMed, whereas the international anchor was derived primarily from the 2025 revised International Association of Pancreatology guideline, supplemented by the 2024 American College of Gastroenterology guideline (1, 3, 16). Public-source items were used to capture lay information-seeking intent and patient-oriented phrasing, while guideline-derived items anchored the benchmark to decision-critical clinical content.

Because the original exploratory browsing logs were not retained as a formal dataset, the public-source retrieval component was documented using a retrospectively reconstructed yet reproducible protocol aligned with the final frozen item set. Retrieval procedures for Google Trends and Baidu Zhidao were finalized and frozen on April 20, 2026, before model testing. Candidate items were consolidated at the level of core intent rather than literal wording by two investigators independently, with disagreements adjudicated by a third investigator. Because the source materials included both Chinese- and English-language content, the benchmark was explicitly designed as an English-language evaluation. Chinese-language search intents and guideline-derived concepts were harmonized through bilingual adaptation and standardized into single-intent, patient-facing, non-case-specific English prompts to improve cross-model comparability. Accordingly, this study evaluated model performance in English-language patient communication rather than Chinese-language or multilingual health-information generation. For source items derived from Chinese-language materials, English translation and linguistic adaptation were performed with support from Bullet Edits Limited by a professional native English-language editor. The translated and edited questions were subsequently reviewed by the investigators to ensure preservation of the original clinical intent, patient-facing phrasing, and cross-model comparability. Questions were further balanced across four task categories: red-flag recognition, mechanism explanation, management decision-making, and recovery guidance. All questions were finalized and frozen before model testing. Detailed retrieval settings, screening criteria, privacy-preserving source handling, and source-to-item mapping are provided in the Supplementary material.

### Large language models and query procedure

Five publicly accessible conversational LLMs were evaluated using the following public-interface version identifiers and release dates: GPT-5.4 Thinking (released on March 5, 2026), DeepSeek-V3.2 (released on December 1, 2025), Gemini 3.1 Pro (released on February 19, 2026), Grok 4.3 (released on April 17, 2026), and Qwen3.6-Max-Preview (released on April 20, 2026). Queries were performed on April 21–22, 2026, through the official publicly accessible web interfaces of the evaluated models. No API access, browser extensions, plug-ins, automated scripts, or third-party aggregation platforms were used.

Each finalized English-language prompt was manually entered into the corresponding model interface in private/incognito mode after signing out of Google and other personal accounts. Every prompt was submitted in a separate new session following deletion of prior conversation history and clearing of browser cache. No follow-up prompting, response regeneration, prompt optimization, or selective response replacement was performed. All models were evaluated under default public-user settings. For the primary analysis, a single response per model per prompt was collected to simulate the default public-user scenario in which a patient enters a health-related question once and receives one immediate answer. This design was selected to preserve a standardized cross-sectional public-interface snapshot across models within a narrow query window. However, because LLM outputs may vary across repeated generations and public-interface updates, a single-generation design cannot fully characterize within-model stochasticity or temporal drift.

### Response preprocessing and blinding

Before evaluation, all responses were transferred into a standardized scoring template and assigned anonymized response codes. Model names, interface labels, logos, timestamps, and other identifying information were removed before scoring. Raters were blinded to model identity and response-generation order. However, because LLMs may differ in writing style, formatting, wording patterns, and list structures, complete elimination of potential stylistic recognition could not be ensured. Two licensed Chinese gastroenterologists, each with 10 years of clinical experience, independently evaluated the anonymized responses while remaining blinded to model source. Before formal assessment, the raters reviewed the scoring instruments and rubric definitions to promote consistent interpretation. Discrepant ratings were adjudicated at the item level by a third gastroenterologist. The third reviewer independently reviewed the anonymized model response, the original benchmark prompt, and the two initial item-level ratings, and assigned the final adjudicated item score. Final scores were not determined by consensus discussion or majority vote. Total instrument scores used for descriptive and inferential analyses were calculated from the adjudicated item-level scores, whereas inter-rater reliability estimates were calculated from the two original independent ratings before adjudication.

### Outcome measures

Response quality and transparency-related features were assessed using four established instruments: DISCERN, EQIP, GQS, and JAMA Benchmark Criteria. DISCERN was scored from 16 to 80, with higher scores indicating better reliability and overall quality of health information. EQIP was reported as a percentage score from 0 to 100%, with higher percentages indicating better patient-information quality. GQS was scored from 1 to 5, with higher scores indicating better overall information quality. The JAMA benchmark was scored from 0 to 4 according to visible response-level indicators of authorship, attribution, disclosure, and currency; higher scores indicated more visible transparency-related signals (17–19). In this study, the JAMA benchmark was applied as a structured proxy for visible transparency-related features within the response text rather than as a direct measure of factual accuracy, guideline concordance, or clinical safety. Detailed scoring instruments and interpretation criteria are provided in Supplementary Table S4.

In response to the need to distinguish informational quality from clinical safety, we added a structured response-level safety analysis. Each anonymized response was evaluated for factual inaccuracy, clinical hallucination, and clinical safety-risk severity using a predefined 0–3 rubric. Factual inaccuracy referred to clinically incorrect, misleading, or guideline-discordant statements. Clinical hallucination referred to fabricated clinical claims, unsupported certainty, non-verifiable evidence claims, or guideline-inconsistent recommendations presented as reliable information. Clinical safety risk was graded according to the plausibility that the response could mislead patient behavior, delay urgent medical evaluation, encourage inappropriate self-management, or promote incorrect treatment decisions. These safety-related outcomes were analyzed separately from DISCERN, EQIP, GQS, JAMA benchmark scores, and readability metrics. Detailed safety scoring criteria are provided in Supplementary Table S9.

### Readability assessment

Because all finalized prompts and model outputs were generated in English, only English-language readability formulas were applied. Accordingly, the readability findings should be interpreted as reflecting English-language patient-facing outputs and should not be directly generalized to Chinese-language or multilingual patient education. Readability was evaluated using the Automated Readability Index (ARI), Flesch Reading Ease Score (FRES), Gunning Fog Index (GFI), Flesch–Kincaid Grade Level (FKGL), Coleman-Liau Index (CLI), and Simple Measure of Gobbledygook (SMOG), calculated using the online tool readabilityformulas.com. The same calculator was applied to all responses to maintain internal consistency across models. Consistent with prior patient-education readability studies, target readability thresholds were defined as FRES ≥ 80.0 and ≤ 6 for the remaining five formulas (20, 21).

### Statistical analysis

All statistical analyses were performed using IBM SPSS Statistics 29.0 and R version 4.3.2. Visualizations were generated using Flourish. Descriptive data are presented as mean ± standard deviation. For the added safety-related outcomes, inter-rater agreement was assessed using weighted Cohen’s kappa for ordinal severity ratings and Cohen’s kappa for binary indicators of any factual inaccuracy, any clinical hallucination, and any clinical safety risk. Model-level safety findings were summarized descriptively as counts and percentages. Because no moderate- or high-risk clinical safety event was identified, inferential between-model safety comparisons were not emphasized. Agreement analyses were based on the two physicians’ initial independent ratings rather than adjudicated final scores. Because each question was evaluated across all five models, overall between-model differences were analyzed using the Friedman test. When overall significance was observed, *post hoc* pairwise comparisons were conducted using paired Wilcoxon signed-rank tests with Holm-Bonferroni correction. For the omnibus Friedman tests across the prespecified quality/transparency-related and readability endpoints, both raw *p* values and Holm-adjusted p values across endpoints were reported. For each post-hoc paired Wilcoxon signed-rank comparison, pairwise effect sizes were calculated using matched-pairs rank-biserial correlation, with 95% confidence intervals estimated by nonparametric bootstrap resampling at the question level. Paired median differences were also reported to provide an interpretable estimate of the direction and magnitude of between-model differences. Positive rank-biserial correlation values indicate higher raw scores for the first-listed model; for ARI, GFI, FKGL, CLI, and SMOG, higher raw scores indicate greater reading difficulty, whereas higher FRES values indicate easier reading. Readability scores were compared against predefined sixth-grade thresholds using the Wilcoxon signed-rank test. Statistical significance was defined as a two-sided *p* < 0.05. To assess the potential impact of within-model output variability on ranking stability, we added an exploratory repeated-generation sensitivity analysis. Six representative prompts were selected from the original benchmark set, covering emergency warning signs, common causes, antibiotic use, ERCP indications, refeeding, and post-recovery management of biliary or metabolic causes. For each selected prompt, two additional independent generations were obtained from each of the five models under the same standardized public-interface conditions, yielding 60 additional responses. The additional responses were scored using the same instruments and adjudication workflow as the primary analysis. Model-level rankings from the original single-generation subset were compared with rankings derived from the mean of the two additional generations using Spearman rank correlation for DISCERN, EQIP, JAMA benchmark score, and GQS. After formal scoring and adjudication had been completed and the original ratings had been locked, an exploratory post-hoc model-identity guessing subset sensitivity analysis was conducted to assess potential stylistic unblinding. Because the analysis was added after the original scoring workflow, a balanced subset rather than the full response set was used. Four benchmark questions were selected to represent the four predefined task categories: red-flag recognition, mechanism explanation, management decision-making, and recovery guidance. The same four questions were sampled across all five evaluated models, yielding 20 anonymized responses in total, with four responses per model. The two original physician raters independently re-reviewed these anonymized response texts and selected one presumed source model from the five candidate models for each response. Because five models were evaluated, the prespecified random-guess probability was 20%. Guessing accuracy was summarized for each rater separately and in pooled form, with 95% confidence intervals, and compared with the 20% chance level using exact binomial testing.

### Ethical considerations

This study did not involve human participants, direct interaction with individuals, identifiable private information, or patient-level data. The analysis relied exclusively on publicly available search-trend data, publicly visible title-level online question phrasing, published clinical guidelines, and model-generated text. Under institutional policy, formal ethics review was not required because no human participants or identifiable private information were involved, and informed consent was therefore not applicable.

## Results

### Benchmark composition and response dataset

The final benchmark comprised 24 English-language, single-intent, patient-facing questions on acute pancreatitis, balanced across six prespecified clinical domains and four source categories. The questions addressed symptoms and thresholds for seeking care, causes and recurrence, diagnosis and severity assessment, early treatment, nutrition and inpatient management, and complications and post-discharge management. The complete question set is presented in Table 1. Detailed source retrieval procedures and source-to-item mapping are provided in Supplementary Appendix 1 and Supplementary Table S1.

**Table 1:** Final benchmark questions for evaluating large language models in patient-facing acute pancreatitis information.

Item ID	Final benchmark question
Q1	What warning signs in acute pancreatitis mean I should go to the hospital right away?
Q2	In acute pancreatitis, when is it no longer safe to stay at home and keep observing?
Q3	Why is it important to assess the severity of acute pancreatitis early?
Q4	What changes may suggest that acute pancreatitis is getting worse?
Q5	What are the most common causes of acute pancreatitis?
Q6	Why can pancreatitis come back even after it starts to get better?
Q7	Why can gallstones cause acute pancreatitis?
Q8	How can the risk of recurrence be reduced after discharge for acute pancreatitis?
Q9	How do doctors usually determine whether someone has acute pancreatitis?
Q10	Is a CT scan always needed to diagnose pancreatitis?
Q11	Why can the severity of acute pancreatitis not be judged from just one test?
Q12	What are the main differences between mild and severe acute pancreatitis?
Q13	Why does fluid treatment in early acute pancreatitis need to be adjusted according to the patient’s condition?
Q14	Do all patients with acute pancreatitis need antibiotics?
Q15	What are the key parts of early treatment for acute pancreatitis?
Q16	In gallstone-related acute pancreatitis, when is ERCP needed?
Q17	Why should patients with acute pancreatitis not eat freely in the early stage?
Q18	When can eating be started again in acute pancreatitis?
Q19	What do doctors mainly consider when deciding whether eating can be resumed in acute pancreatitis?
Q20	Why is early enteral nutrition recommended in acute pancreatitis?
Q21	After recovery from acute pancreatitis, what problems may mean that further follow-up is still needed?
Q22	After discharge for pancreatitis, what problems should prompt an early follow-up visit?
Q23	After apparent recovery from acute pancreatitis, why is it still important to address biliary or metabolic causes?
Q24	After discharge for acute pancreatitis, what symptoms suggest that urgent medical reassessment is needed?

The final benchmark comprised 24 English-language, single-intent, patient-facing questions. Detailed clinical domains, source categories, task types, search and guideline anchors, and the rationale for item selection and retention are provided in Supplementary Table S1.

**Figure 1:**
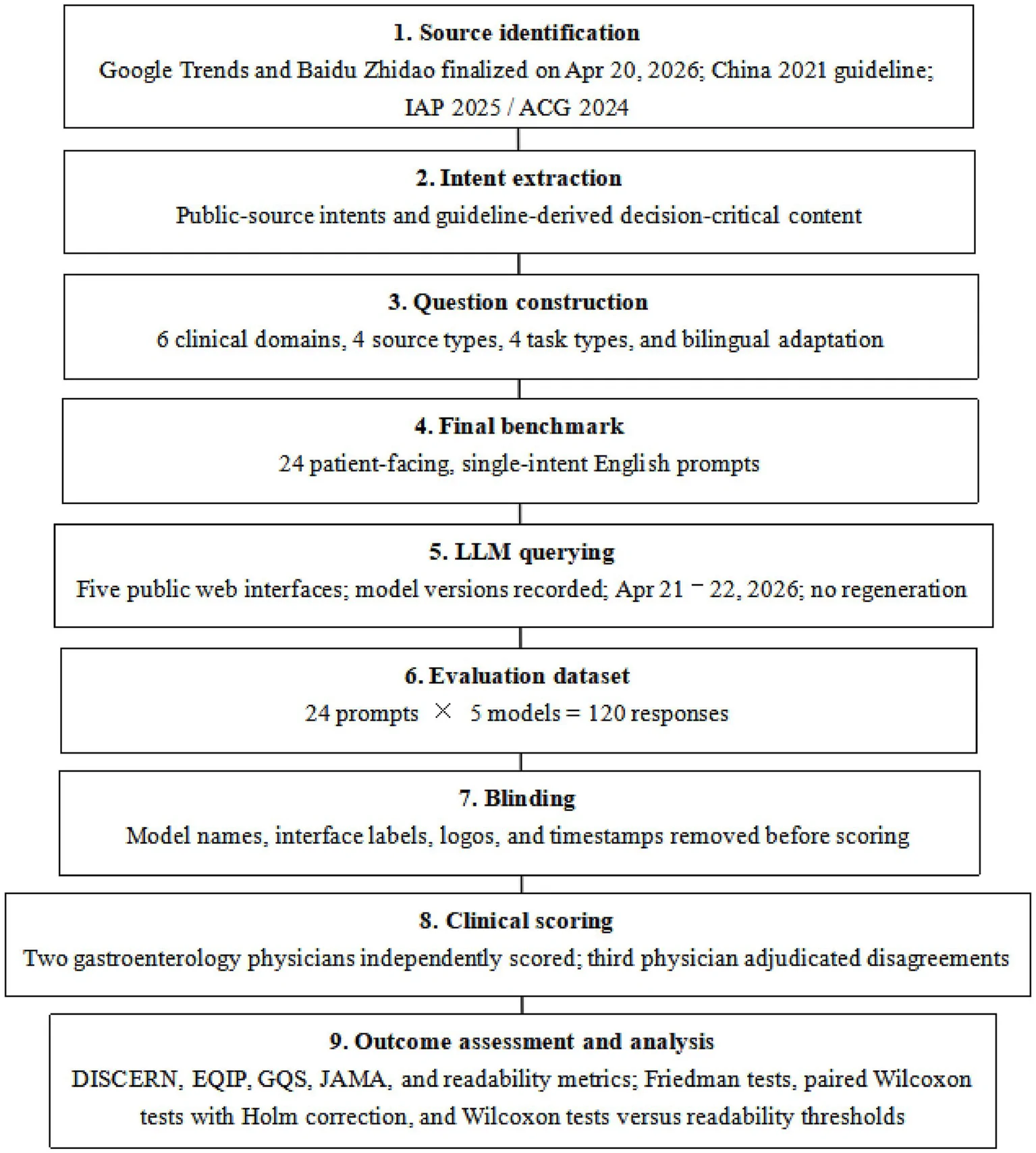
Question development, model querying, and evaluation workflow. The benchmark was constructed from public-source search intents and guideline-derived decision-critical content and standardized into 24 patient-facing, single-intent English prompts across six clinical domains and four task types. Each prompt was submitted once to five publicly accessible LLM web interfaces, yielding 120 responses. Model-identifying information was removed prior to blinded clinical assessment. Outcomes included DISCERN, EQIP, GQS, JAMA benchmark scores, and readability metrics, followed by repeated-measures statistical analyses at the question level.

The overall study workflow, including question development, model querying, blinded evaluation, readability assessment, and statistical analysis, is summarized in Figure 1. Each of the 24 questions was submitted once to each of the five evaluated LLMs, yielding 120 responses for analysis. In the exploratory repeated-generation sensitivity analysis, six representative prompts were re-submitted twice to each of the five evaluated models, yielding 60 additional responses. Compared with the original single-generation results for the same six prompts, model-level rank correlations were 1.00 for DISCERN, 0.89 for EQIP, 1.00 for JAMA benchmark score, and 0.89 for GQS. Grok 4.3 remained the highest-ranked model for DISCERN, EQIP, and JAMA benchmark scores and was tied for the highest GQS score in the repeated-generation subset. Qwen3.6-Max-Preview remained within the top tier. Minor reordering was observed among intermediate-ranked models, indicating that small between-model differences should be interpreted cautiously. Detailed repeated-generation sensitivity-analysis results are provided in Supplementary Table S12. The complete original text of these 120 model-generated responses is provided in Supplementary Appendix 4. The blinded scoring workflow and rubric safeguards for generalized question stems are described in Supplementary Appendix 2 and Supplementary Table S2. Readability and statistical procedures, together with model access and query conditions, are summarized in Supplementary Appendix 3 and Supplementary Table S3. Scoring instruments and interpretation criteria are presented in Supplementary Table S4.

Pre-adjudication inter-rater agreement was excellent for the DISCERN total score (ICC[A,1] = 0.918, 95% CI 0.892–0.935), moderate for the EQIP percentage score (ICC[A,1] = 0.643, 95% CI 0.518–0.729), almost perfect for GQS (quadratic-weighted *κ* = 0.881, 95% CI 0.777–0.962), and almost perfect for the JAMA benchmark score (quadratic-weighted κ = 0.921, 95% CI 0.863–0.966). Agreement was lower for EQIP than for the other evaluated outcomes, as detailed in Supplementary Table S5. In the exploratory post-hoc model-identity guessing subset sensitivity analysis, 20 anonymized responses were evaluated, including four responses from each model. The observed model-identity guessing accuracy was 5/20 for Rater 1 (25.0%; 95% CI, 8.7–49.1%; exact binomial *p* = 0.576) and 6/20 for Rater 2 (30.0%; 95% CI, 11.9–54.3%; exact binomial *p* = 0.265). The pooled guessing accuracy was 11/40 (27.5%; 95% CI, 14.6–43.9%), which was not significantly higher than the prespecified random-guess probability of 20% (exact binomial *p* = 0.237). These findings did not provide consistent evidence that raters could reliably identify model sources from writing style alone in the selected subset, although partial stylistic recognition could not be completely excluded. Detailed model-identity guessing results are provided in Supplementary Table S11.

### Quality and transparency-related scores

Quality and transparency-related scores differed across the five LLMs. Across the 24 questions, Grok 4.3 achieved the highest mean DISCERN score (55.71 ± 6.94), GQS score (4.42 ± 0.50), EQIP percentage score (80.83 ± 6.20), and JAMA benchmark score (1.08 ± 0.83), indicating the strongest informational quality profile according to the predefined quality and transparency-related instruments rather than direct evidence of clinical safety superiority. Qwen3.6-Max-Preview and DeepSeek-V3.2 demonstrated intermediate overall quality performance, with DISCERN scores of 52.12 ± 7.86 and 51.00 ± 6.43, respectively. GPT-5.4 Thinking produced the lowest mean DISCERN score (42.62 ± 6.27), whereas Gemini 3.1 Pro showed intermediate DISCERN and EQIP performance but the lowest mean GQS score (3.88 ± 0.34). Descriptive statistics for all quality and transparency-related outcomes are presented in Table 2.

**Table 2:** Quality and transparency-related scores by model (mean ± SD, *n* = 24 questions).

Model	DISCERN	GQS	EQIP (%)	JAMA
GPT-5.4 Thinking	42.62 ± 6.27	3.96 ± 0.36	71.25 ± 6.80	0.25 ± 0.53
DeepSeek-V3.2	51.00 ± 6.43	4.08 ± 0.50	73.75 ± 6.47	0.12 ± 0.34
Gemini 3.1 Pro	46.21 ± 6.61	3.88 ± 0.34	72.50 ± 6.59	0.12 ± 0.34
Grok 4.3	55.71 ± 6.94	4.42 ± 0.50	80.83 ± 6.20	1.08 ± 0.83
Qwen3.6-Max-Preview	52.12 ± 7.86	4.25 ± 0.53	76.88 ± 5.48	0.62 ± 0.82

EQIP is reported as a percentage score. The JAMA benchmark was used as a proxy for visible transparency-related features rather than as a direct measure of factual accuracy or clinical safety.

**Figure 2:**
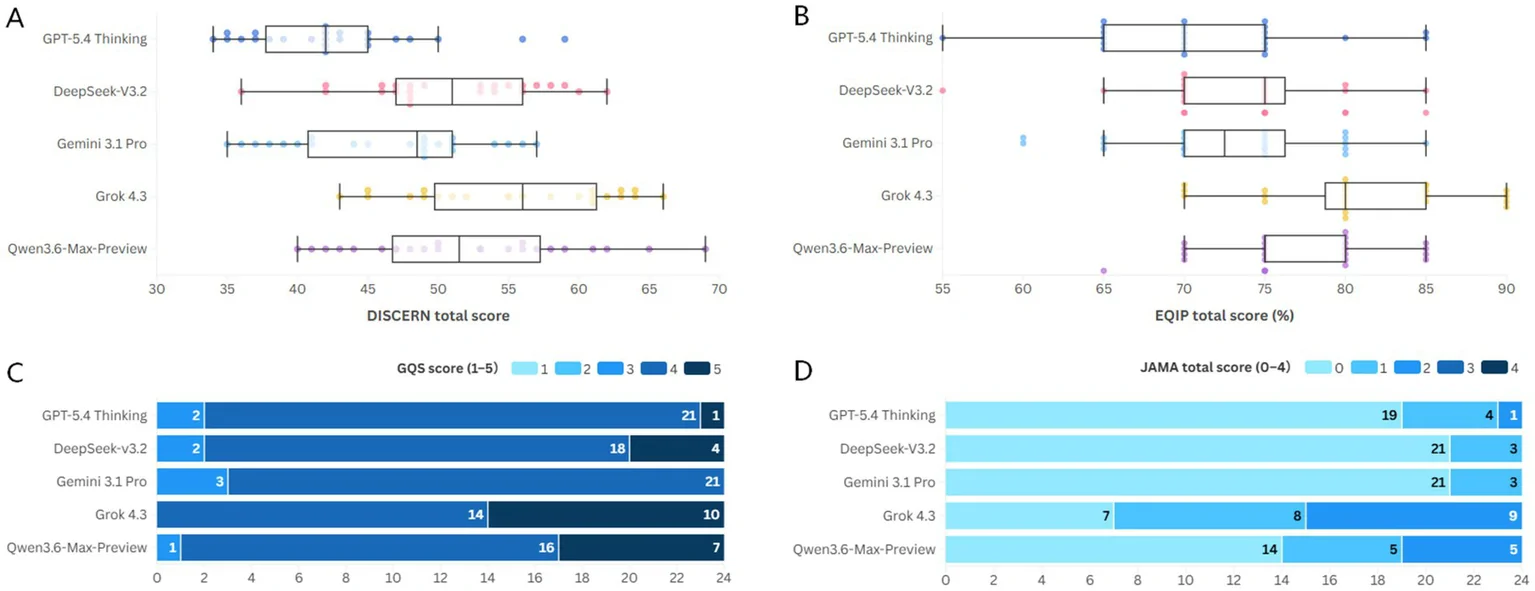
Distribution of quality and transparency-related scores across five large language models. Panels **(A,B)** display box-and-whisker plots with individual response-level data points for DISCERN scores and EQIP percentage scores, respectively. Panels **(C,D)** present stacked distributions of Global Quality Scale (GQS) scores and Journal of the American Medical Association (JAMA) benchmark scores, respectively. Scores were derived across the 24 acute pancreatitis benchmark questions for each model. Higher DISCERN, EQIP, and GQS scores indicate greater informational reliability, patient-information quality, and overall quality, respectively, whereas higher JAMA benchmark scores reflect a greater presence of visible transparency-related features.

Response-level distributions of quality and transparency-related scores are shown in Figure 2. Grok 4.3 generally demonstrated higher DISCERN, EQIP, and GQS score distributions than the other models, although substantial overlap remained across models. In contrast, JAMA benchmark scores were consistently low across all models, indicating limited presence of visible transparency-related features such as authorship, attribution, disclosure, and currency within unprompted response text. Even the highest mean JAMA score, observed for Grok 4.3, remained close to 1 on the 0–4 scale. The repeated-generation sensitivity analysis supported the broad stability of the main quality-ranking pattern in the selected subset, while also showing that minor rank changes may occur among closely performing intermediate models.

### Factual accuracy, clinical hallucination, and safety-risk analysis

Model-level distributions of safety-related outcomes are summarized in Table 3. The table presents counts and percentages for responses with any factual inaccuracy, any clinical hallucination, and any clinical safety risk, together with the corresponding 0/1/2/3 severity distributions. All clinical safety-risk signals were adjudicated as score 1 (low risk) under the predefined clinician-rated rubric, and no response was adjudicated as having moderate- or high-risk clinical safety risk.

**Table 3:** Factual inaccuracies, clinical hallucinations, and clinical safety-risk signals by model.

Model	Any factual inaccuracy, *n* (%)	Any clinical hallucination, *n* (%)	Any clinical safety risk, *n* (%)	Factual inaccuracy 0/1/2/3	Hallucination 0/1/2/3	Safety risk 0/1/2/3
GPT-5.4 Thinking	0/24 (0.0)	0/24 (0.0)	0/24 (0.0)	24/0/0/0	24/0/0/0	24/0/0/0
DeepSeek-V3.2	9/24 (37.5)	9/24 (37.5)	4/24 (16.7)	15/5/4/0	15/5/4/0	20/4/0/0
Gemini 3.1 Pro	1/24 (4.2)	1/24 (4.2)	1/24 (4.2)	23/0/1/0	23/1/0/0	23/1/0/0
Grok 4.3	1/24 (4.2)	1/24 (4.2)	1/24 (4.2)	23/1/0/0	23/1/0/0	23/1/0/0
Qwen3.6-Max-Preview	5/24 (20.8)	5/24 (20.8)	1/24 (4.2)	19/5/0/0	19/5/0/0	23/1/0/0
Overall	16/120 (13.3)	16/120 (13.3)	7/120 (5.8)	104/11/5/0	104/12/4/0	113/7/0/0

Values are adjudicated final ratings. For each severity distribution, values indicate counts for scores 0/1/2/3. All clinical safety-risk signals were adjudicated as score 1 (low risk) under the predefined clinician-rated rubric; this classification reflects expert-assessed potential risk rather than observed patient behavior. No response was adjudicated as having moderate- or high-risk clinical safety risk.

In the added safety analysis, factual inaccuracies were identified in 16 of 120 responses (13.3%), including 11 low-severity and 5 moderate-severity events. Clinical hallucinations were also identified in 16 responses (13.3%), including 12 low-severity and 4 moderate-severity events. Clinical safety-risk signals were identified in 7 responses (5.8%), all of which were adjudicated as score 1 (low risk) under the predefined clinician-rated rubric. No response was adjudicated as having high-risk factual inaccuracy, high-risk clinical hallucination, or moderate-to-high clinical safety risk.

At the model level, GPT-5.4 Thinking had no adjudicated factual inaccuracies, clinical hallucinations, or safety-risk signals. DeepSeek-V3.2 had the highest number of factual inaccuracies and clinical hallucinations, each occurring in 9 of 24 responses (37.5%), and the highest number of clinical safety-risk signals, occurring in 4 of 24 responses (16.7%). Gemini 3.1 Pro and Grok 4.3 each had one response with factual inaccuracy, clinical hallucination, and a clinician-adjudicated score 1 (low-risk) safety signal. Qwen3.6-Max-Preview had factual inaccuracies and clinical hallucinations in 5 of 24 responses (20.8%) and one clinician-adjudicated score 1 (low-risk) safety signal.

The antibiotic-related item, ‘Do all patients with acute pancreatitis need antibiotics?’, did not receive any adjudicated factual inaccuracy, clinical hallucination, or clinical safety-risk score for any model, including Grok 4.3. Thus, the added safety analysis did not identify any response advising unsupervised antibiotic use at home for mild acute pancreatitis.

Pre-adjudication inter-rater agreement for the newly added safety-related ratings was moderate to substantial. Weighted kappa values were 0.619 (95% CI, 0.49–0.75) for factual inaccuracy severity, 0.572 (95% CI, 0.44–0.70) for clinical hallucination severity, and 0.621 (95% CI, 0.49–0.75) for clinical safety-risk severity. For binary indicators, Cohen’s kappa values were 0.592 (95% CI, 0.46–0.72) for any factual inaccuracy, 0.606 (95% CI, 0.48–0.73) for any clinical hallucination, and 0.744 (95% CI, 0.62–0.87) for any clinical safety risk.

An exploratory stratified descriptive analysis by clinical risk tier is provided in Supplementary Table S10; this analysis grouped red-flag recognition and management-decision items as high-stakes/decision-critical questions and mechanism-explanation and recovery-guidance items as lower-immediacy educational/recovery questions.

### Readability scores

Readability analyses demonstrated that all models generated text substantially exceeding the predefined patient-education readability thresholds. DeepSeek-V3.2 showed the most favorable overall readability profile across all six formulas, achieving the lowest ARI (12.01 ± 2.26), GFI (12.59 ± 2.01), FKGL (11.05 ± 2.11), CLI (12.76 ± 2.02), and SMOG (10.29 ± 1.69) scores, together with the highest FRES (44.42 ± 12.27). GPT-5.4 Thinking demonstrated the second most favorable readability profile for several indices, including FRES (34.08 ± 12.68), GFI (13.01 ± 1.86), and SMOG (11.25 ± 1.90). In contrast, Grok 4.3 and Qwen3.6-Max-Preview generally produced more difficult-to-read text, with particularly low FRES values of 16.92 ± 8.52 and 16.25 ± 8.14, respectively. Complete readability results are presented in Table 4.

**Table 4:** Readability metrics by model (mean ± SD, *n* = 24 questions).

Model	ARI	FRES	GFI	FKGL	CLI	SMOG
GPT-5.4 Thinking	14.47 ± 2.80	34.08 ± 12.68	13.01 ± 1.86	13.30 ± 2.48	14.28 ± 1.89	11.25 ± 1.90
DeepSeek-V3.2	12.01 ± 2.26	44.42 ± 12.27	12.59 ± 2.01	11.05 ± 2.11	12.76 ± 2.02	10.29 ± 1.69
Gemini 3.1 Pro	14.78 ± 1.89	30.42 ± 9.74	14.42 ± 1.59	13.78 ± 1.75	14.93 ± 1.54	12.25 ± 1.27
Grok 4.3	17.73 ± 1.92	16.92 ± 8.52	15.74 ± 1.38	15.62 ± 1.51	17.80 ± 1.52	13.29 ± 1.28
Qwen3.6-Max-Preview	15.05 ± 1.49	16.25 ± 8.14	15.86 ± 1.49	14.23 ± 1.35	17.85 ± 1.56	11.56 ± 0.91
Recommended threshold	≤6	≥80	≤6	≤6	≤6	≤6

For FRES, higher values indicate easier readability, with a recommended threshold of ≥ 80. For ARI, GFI, FKGL, CLI, and SMOG, lower values indicate easier readability, with a recommended threshold of ≤6.

**Figure 3:**
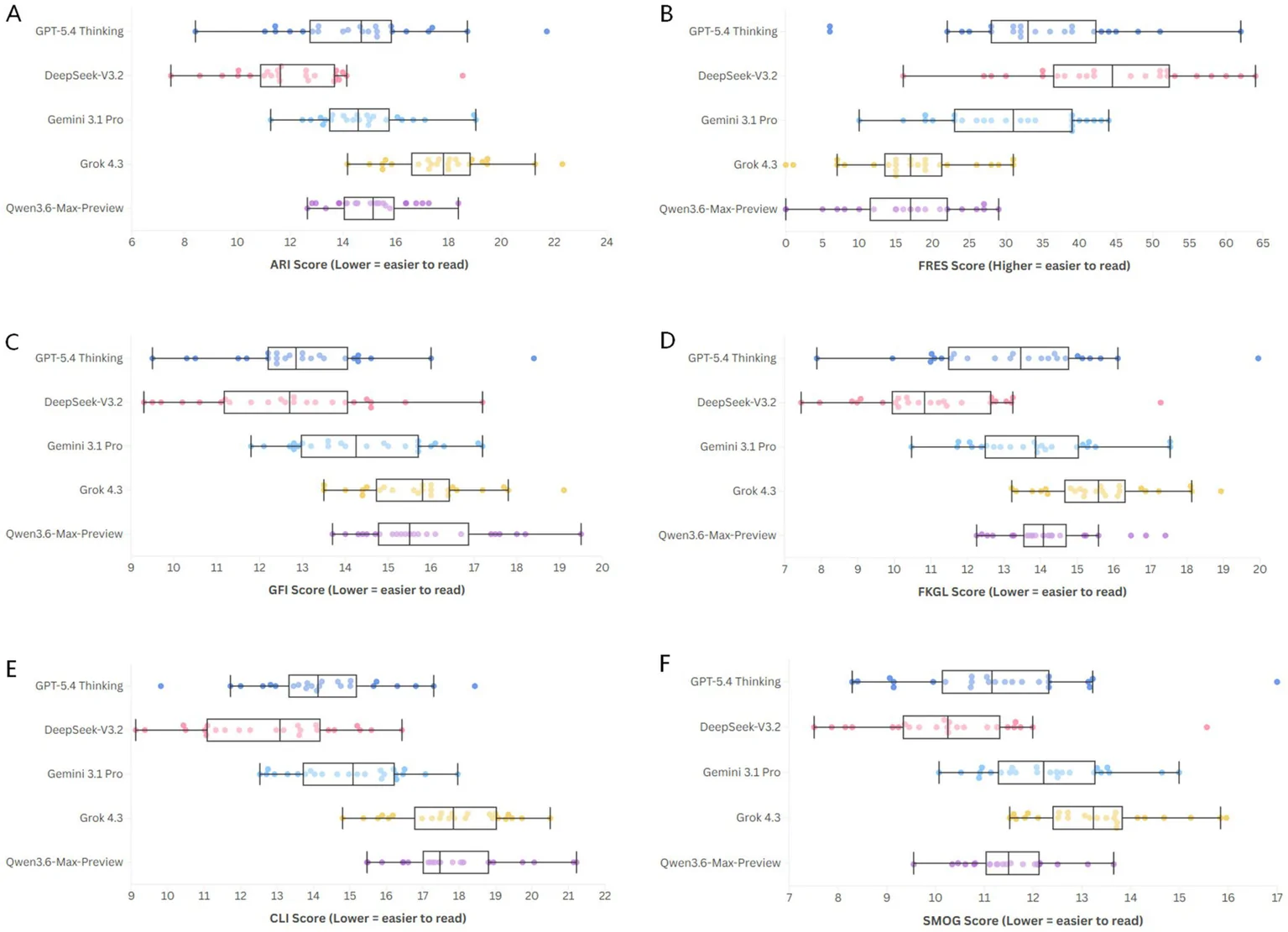
Distribution of readability metrics across five large language models. Box-and-whisker plots with individual response-level data points illustrate readability score distributions across the 24 acute pancreatitis benchmark questions for each model. **(A)**, Automated Readability Index (ARI). **(B)**, Flesch Reading Ease Score (FRES). **(C)**, Gunning Fog Index (GFI). **(D)** Flesch–Kincaid Grade Level (FKGL). **(E)** Simple Measure of Gobbledygook (SMOG). **(F)** Coleman-Liau Index (CLI). For ARI, GFI, FKGL, CLI, and SMOG, lower scores indicate easier readability, whereas for FRES, higher scores indicate easier readability. Prespecified patient-education thresholds were defined as FRES ≥80 and ≤6 for the remaining five grade-level indices.

Response-level distributions for the six readability formulas are shown in Figure 3. Across all five models, mean FRES values remained substantially below the recommended threshold of ≥ 80, whereas mean ARI, GFI, FKGL, CLI, and SMOG values consistently exceeded the recommended threshold of ≤ 6. Examination of the raw readability data at the individual-response level further demonstrated that none of the 120 outputs satisfied all six predefined readability targets.

### Normalized performance profiles

**Figure 4:**
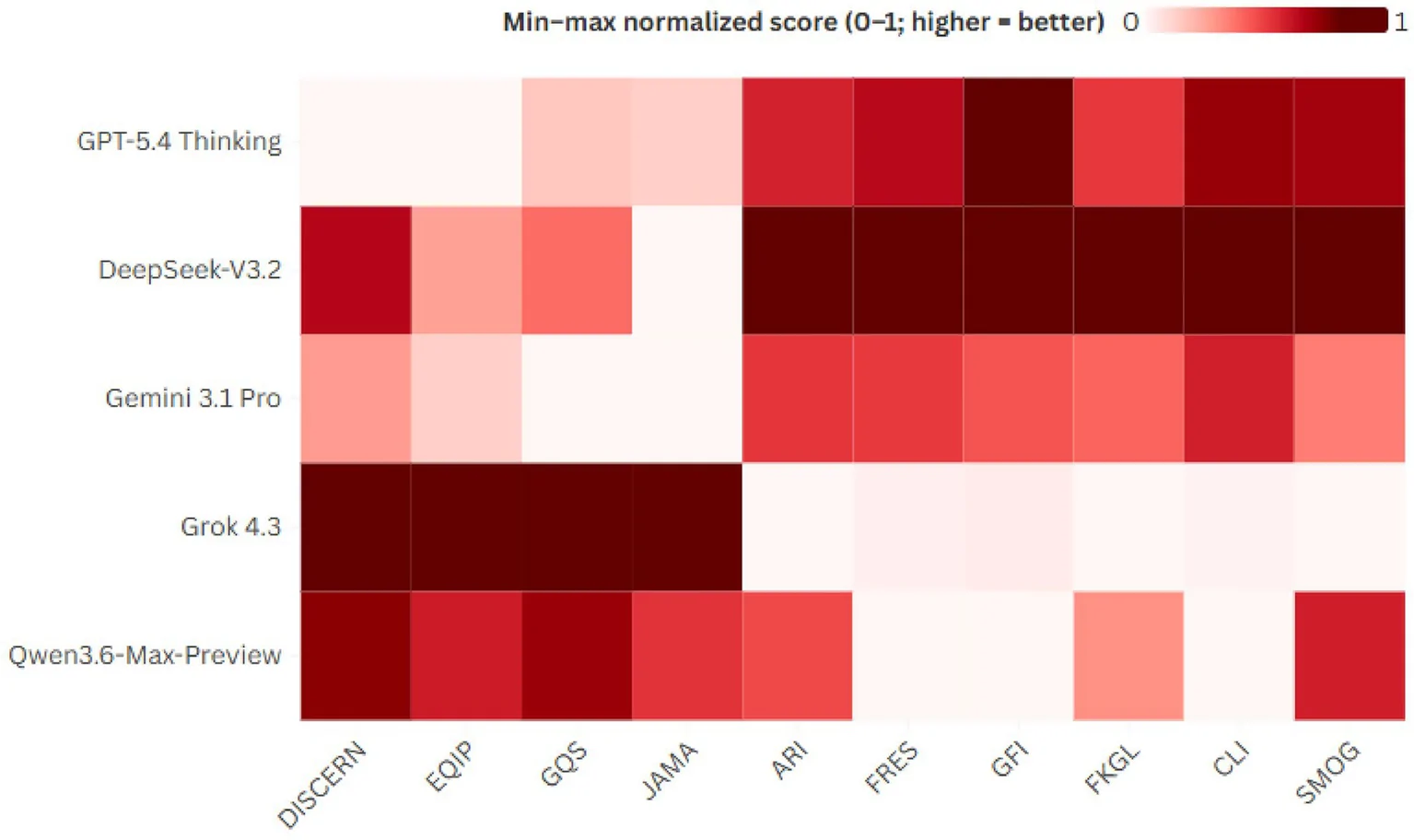
Normalized performance heatmap across quality/transparency-related and readability metrics. Rows represent the five evaluated large language models, and columns represent quality/transparency-related and readability metrics. Values were min–max normalized within each metric to a 0–1 scale. For DISCERN, EQIP, GQS, JAMA, and FRES, higher raw values indicate better performance. For ARI, GFI, FKGL, CLI, and SMOG, values were reverse-coded prior to normalization so that higher normalized scores consistently represent better relative performance. Darker shading indicates better relative performance within each metric. For readability metrics, higher normalized values therefore reflect easier readability after reverse-coding.

To facilitate comparison across heterogeneous outcome scales, all quality, transparency-related, and readability metrics underwent min–max normalization. For readability indices in which lower raw values corresponded to easier reading, scores were reverse-coded before normalization so that higher normalized values consistently reflected better relative performance. The normalized heatmap is presented in Figure 4.

The normalized heatmap demonstrated divergent performance patterns across models, with higher normalized quality scores not consistently associated with higher normalized readability scores. Grok 4.3 achieved the highest normalized values for most quality and transparency-related outcomes, whereas its normalized readability performance was comparatively poorer. DeepSeek-V3.2 demonstrated the most favorable normalized readability profile, together with intermediate normalized quality performance. Qwen3.6-Max-Preview showed relatively higher normalized quality and transparency-related values than several other models but lower normalized readability values, particularly for FRES, GFI, and CLI. GPT-5.4 Thinking and Gemini 3.1 Pro generally demonstrated lower or intermediate normalized performance depending on the evaluated metric.

### Overall between-model differences

Overall between-model differences were statistically significant across all quality, transparency-related, and readability outcomes. Friedman tests demonstrated significant between-model differences for DISCERN (χ2 = 59.183, Kendall’s *W* = 0.616, Holm-adjusted *p* < 0.0001), GQS (χ2 = 20.686, *W* = 0.215, Holm-adjusted *p* < 0.001), EQIP percentage score (χ2 = 28.750, *W* = 0.299, Holm-adjusted *p* < 0.0001), and JAMA benchmark score (χ2 = 37.889, *W* = 0.395, Holm-adjusted *p* < 0.0001). Significant between-model differences were likewise observed across all readability metrics, with Kendall’s *W* values ranging from 0.510 for SMOG to 0.844 for CLI. Full Friedman test statistics, effect-size estimates, raw *p* values, and Holm-adjusted *p* values are provided in Table 5.

**Table 5:** Overall between-model differences across quality/transparency-related and readability metrics.

Metric	Friedman χ^2^	df	Kendall’s *W*	*P* (raw)	*P* (Holm)
DISCERN	59.183	4	0.616	<0.0001	<0.0001
GQS	20.686	4	0.215	<0.001	<0.001
EQIP (%)	28.750	4	0.299	<0.0001	<0.0001
JAMA	37.889	4	0.395	<0.0001	<0.0001
ARI	58.067	4	0.605	<0.0001	<0.0001
FRES	71.175	4	0.741	<0.0001	<0.0001
GFI	61.476	4	0.640	<0.0001	<0.0001
FKGL	50.497	4	0.526	<0.0001	<0.0001
CLI	81.067	4	0.844	<0.0001	<0.0001
SMOG	48.933	4	0.510	<0.0001	<0.0001

Overall between-model differences were assessed using Friedman tests for repeated measures at the question level. Holm-adjusted *P*-values were applied for multiple comparisons across the listed endpoints. Kendall’s *W* is reported as the corresponding effect-size measure.

### Pairwise model comparisons

**Figure 5:**
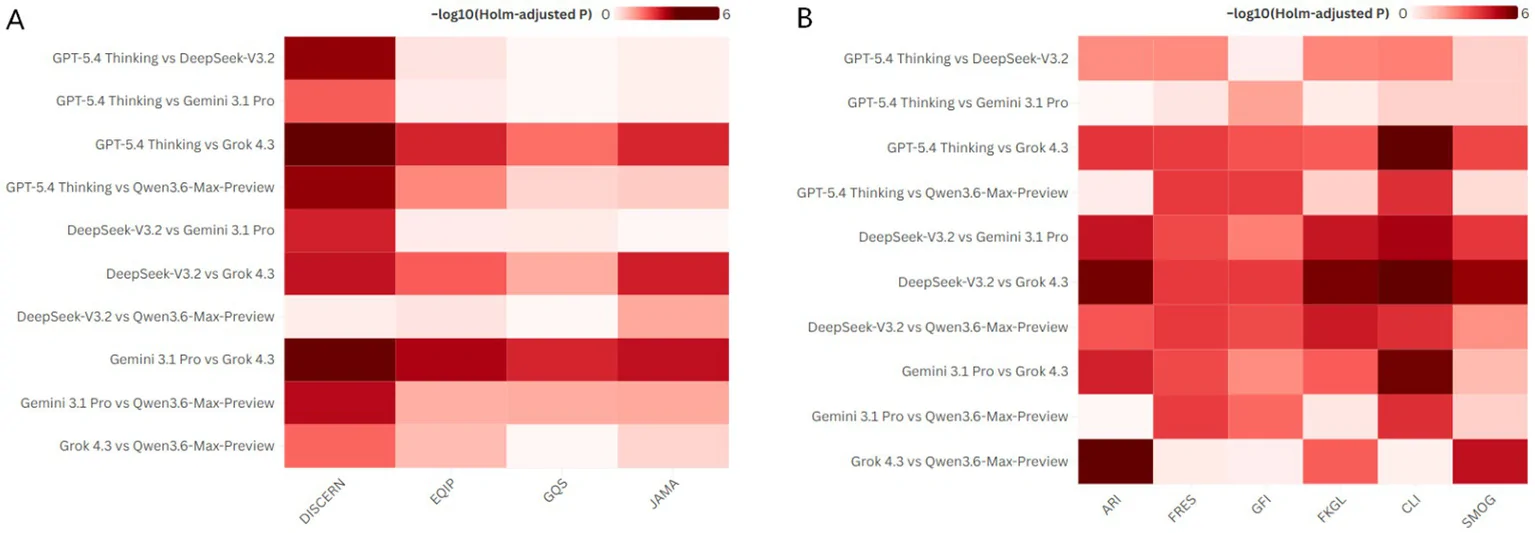
Pairwise model differences across quality/transparency-related and readability metrics. Heatmap values represent −log_10_(Holm-adjusted *p* values) derived from paired Wilcoxon signed-rank comparisons following significant Friedman tests. **(A)** Pairwise differences for quality/transparency-related metrics, including DISCERN, EQIP percentage score, Global Quality Scale (GQS), and Journal of the American Medical Association (JAMA) benchmark score. **(B)** Pairwise differences for readability metrics, including Automated Readability Index (ARI), Flesch Reading Ease Score (FRES), Gunning Fog Index (GFI), Flesch–Kincaid Grade Level (FKGL), Coleman-Liau Index (CLI), and Simple Measure of Gobbledygook (SMOG). Darker shading indicates stronger statistical evidence for between-model differences rather than larger effect sizes.

Pairwise model comparisons following significant Friedman tests are summarized in Figure 5. Detailed post-hoc paired Wilcoxon signed-rank results, including paired median differences, matched-pairs rank-biserial correlations, 95% confidence intervals, raw *p* values, and Holm-adjusted *p* values, are provided in Supplementary Table S6 for quality and transparency-related outcomes and in Supplementary Table S7 for readability outcomes.

For quality and transparency-related outcomes, Grok 4.3 achieved significantly higher DISCERN scores than all other models. It also demonstrated significantly higher EQIP percentage scores than GPT-5.4 Thinking, DeepSeek-V3.2, and Gemini 3.1 Pro, although the difference relative to Qwen3.6-Max-Preview was not statistically significant. For GQS, Grok 4.3 scored significantly higher than GPT-5.4 Thinking and Gemini 3.1 Pro, whereas comparisons with DeepSeek-V3.2 and Qwen3.6-Max-Preview did not reach statistical significance. Regarding JAMA benchmark scores, Grok 4.3 performed significantly better than GPT-5.4 Thinking, DeepSeek-V3.2, and Gemini 3.1 Pro, but not significantly better than Qwen3.6-Max-Preview.

For readability outcomes, DeepSeek-V3.2 generally demonstrated significantly more favorable readability performance than Gemini 3.1 Pro, Grok 4.3, and Qwen3.6-Max-Preview across most or all readability formulas. Relative to GPT-5.4 Thinking, DeepSeek-V3.2 achieved significantly more favorable ARI, FRES, FKGL, and CLI scores, whereas differences in GFI and SMOG were not statistically significant. Grok 4.3 and Qwen3.6-Max-Preview generally produced less readable outputs, although their relative rankings varied depending on the formula applied.

Wilcoxon signed-rank tests comparing model readability scores with predefined readability thresholds demonstrated that all five models differed significantly from recommended patient-education targets in the direction of poorer readability across all six formulas. For ARI, GFI, FKGL, CLI, and SMOG, all model-level distributions were significantly above the ≤ 6 threshold, whereas FRES distributions were significantly below the ≥80 threshold. All exact *p* values were <0.0001, as detailed in Supplementary Table S8.

Collectively, these findings demonstrated a dissociation among informational quality, visible transparency-related signals, readability, and clinical safety. Although several models achieved comparatively higher quality scores, JAMA benchmark scores remained uniformly low across all models, none of the 120 outputs satisfied all predefined readability targets, and the added safety analysis identified a small number of clinician-adjudicated score 1 (low-risk) safety signals. Higher informational quality, therefore, did not necessarily establish stronger visible transparency-related features, patient-education-level readability, factual accuracy, or clinical safety.

## Discussion

### Principal findings

In this cross-sectional benchmark study, five publicly accessible LLMs were evaluated using 24 standardized patient-facing questions on acute pancreatitis spanning six clinical domains and four task categories. Four principal findings emerged. First, model performance differed significantly across quality, transparency-related, and readability outcomes. Grok 4.3 achieved the numerically highest mean DISCERN, EQIP, GQS, and JAMA benchmark scores. However, pairwise analyses demonstrated that its advantage over Qwen3.6-Max-Preview reached statistical significance for DISCERN only, but not for EQIP, GQS, or JAMA benchmark scores. Accordingly, Grok 4.3 should be interpreted as demonstrating the strongest informational quality profile within this benchmark according to the predefined quality and transparency-related instruments, rather than consistent superiority across all quality-related domains or direct clinical safety superiority. Second, visible transparency-related signals remained consistently limited. Even the highest mean JAMA benchmark score remained close to 1 on a 0–4 scale, indicating that authorship, attribution, disclosure, and currency cues were rarely present in unprompted response text. Third, and most clinically relevant for patient education, all models generated responses substantially exceeding predefined patient-education readability thresholds. No individual response satisfied all six predefined readability targets, and all model-level readability distributions differed significantly from recommended thresholds in the direction of poorer readability. This gap remained substantial even for the model with the most favorable readability profile: although DeepSeek-V3.2 achieved the highest mean FRES among the evaluated models, its mean score reached only 44.42, far below the recommended threshold of ≥80.

These findings demonstrate a dissociation among informational quality, visible transparency, and readability. The added safety analysis further underscores that quality scores and safety are related but non-identical constructs. A response may be detailed, coherent, and well structured, thereby receiving higher DISCERN, EQIP, or GQS scores, while still containing a clinically meaningful inaccuracy or unsupported recommendation. Conversely, a less comprehensive response may avoid unsafe advice but provide lower informational quality. The added repeated-generation sensitivity analysis partially addressed concerns about stochastic variation. The main quality-ranking pattern was broadly stable in the selected subset, but the observed minor reordering among intermediate-ranked models indicates that small rank differences should be interpreted cautiously. These findings support the use of the present results as standardized public-interface performance estimates while reinforcing that repeated sampling across larger prompt sets and later timepoints is needed before drawing stronger claims about stable model superiority. Therefore, model rankings derived from quality instruments should be interpreted alongside explicit safety findings, particularly in decision-critical conditions such as acute pancreatitis. Higher quality scores were not consistently associated with improved readability or stronger response-level transparency signals. Because all evaluated prompts and outputs were standardized in English, this dissociation should be interpreted specifically within the context of English-language patient-facing health information. Whether similar quality-readability-transparency relationships would be observed in Chinese-language or multilingual acute pancreatitis responses remains uncertain. Fourth, the added safety analysis showed that factual inaccuracies and clinical hallucinations occurred in a minority of responses, whereas clinical safety-risk signals were uncommon and all were adjudicated as score 1 (low risk) under the predefined clinician-rated rubric. No response was adjudicated as having moderate- or high-risk clinical safety risk. These findings support a clearer distinction between quality-score rankings and safety assessment: Grok 4.3 showed the strongest informational quality profile in this benchmark, but this should not be interpreted as definitive superiority in factual accuracy or clinical safety. The stratified findings further indicate that aggregate model rankings should not be interpreted as uniform superiority across clinical risk contexts, because quality, readability, and safety-related outcomes did not necessarily align within the same model.

One possible explanation is that more detailed and clinically conditional responses may achieve higher quality scores while simultaneously increasing sentence complexity, terminology burden, and reading grade level (22, 23). Conversely, shorter or more plain-language responses may improve readability at the expense of clinical granularity. Because response length, terminology density, and sentence complexity were not formally analyzed as independent mediators, this interpretation should be regarded as hypothesis-generating.

This distinction carries particular clinical relevance in acute pancreatitis, which extends beyond general disease education and frequently involves urgent symptom recognition, early severity assessment, imaging decisions, antibiotic selection, endoscopic intervention, nutritional management, discharge planning, and recurrence prevention (24). Patient-facing information must therefore be not only clinically accurate and comprehensive, but also understandable, actionable, and sufficiently transparent to help users recognize when professional medical evaluation is required (25).

The low JAMA benchmark scores also warrant cautious interpretation. In this study, the JAMA benchmark served as a structured proxy for visible transparency-related features within response text rather than a direct measure of factual accuracy, clinical safety, evidence grounding, or platform-level accountability. The original JAMA benchmark was developed to evaluate accountability in internet-based health information through authorship, attribution, disclosure, and currency. Unprompted conversational LLMs do not inherently provide conventional human authorship statements or conflict-of-interest disclosures. Consequently, low JAMA scores should not be interpreted as evidence of factual inaccuracy. Instead, they indicate that the responses themselves offered limited visible information regarding source attribution, evidentiary basis, disclosure, or update status.

### Comparison with prior work

The present findings align with the broader literature indicating that LLMs may offer meaningful support for patient education while remaining constrained by concerns regarding reliability, transparency, readability, and safe implementation (7, 26). A recent scoping review of LLM applications in patient education suggested that these systems may enhance information access and patient engagement, while emphasizing the need for rigorous evaluation of accuracy, readability, bias, and source attribution before direct patient-facing deployment (10). In the current study, the evaluated models generated coherent and frequently well-structured clinical responses; however, these strengths did not translate into patient-education-level readability or consistent response-level transparency (27, 28).

The readability findings are also consistent with recent investigations of AI-generated medical information across other clinical domains. Studies assessing ChatGPT, Gemini, and Perplexity responses to pain-related and low back pain questions reported that AI-generated outputs frequently exceeded recommended sixth-grade reading levels and required cautious interpretation before patient use (13, 14). Similarly, research examining LLM-assisted simplification of online patient education materials demonstrated that LLMs can improve readability, although performance varies substantially across models and continued human oversight remains necessary to preserve informational completeness and accuracy (15). The present study extends these observations to acute pancreatitis, demonstrating that unprompted outputs from public-interface LLMs may remain substantially above recommended readability thresholds even when certain models achieve comparatively high quality scores.

The clinical context of acute pancreatitis further amplifies the importance of this readability gap. Contemporary guidelines emphasize early severity stratification, selective imaging, appropriate fluid resuscitation, early nutrition when clinically indicated, avoidance of routine antibiotics in the absence of infection, etiology-directed management, and follow-up to reduce recurrence and long-term complications (4, 29). These concepts are clinically nuanced and inherently challenging to convey in simplified language. However, patients with acute pancreatitis exhibit documented health-literacy needs spanning diagnosis, treatment, discharge, recovery, and recurrence prevention (5). Consequently, the central challenge extends beyond whether LLMs can reference guideline-relevant concepts to whether they can communicate such information in a manner that patients can readily understand and appropriately act upon.

### Strengths and implications

This study has several strengths. First, the benchmark was developed using a domain-balanced and source-balanced framework rather than relying on frequency-weighted public queries alone. This design enabled inclusion of both common information-seeking questions and lower-frequency, high-risk, decision-critical topics, including deterioration recognition, triage, timing of ERCP, antibiotic use, refeeding strategies, recurrence prevention, and post-discharge reassessment. Second, all prompts were standardized into English, single-intent, patient-facing questions prior to model evaluation, thereby enhancing cross-model comparability. Third, the analysis incorporated blinded physician scoring, scale-appropriate agreement metrics, multiple validated quality instruments, six established readability formulas, and repeated-measures statistical testing at the question level.

The findings have important implications for AI-assisted patient education. Outputs from current public-interface LLMs should not be considered directly deployable patient education materials solely on the basis of fluency, comprehensiveness, or structural coherence. In this study, the model demonstrating the strongest overall quality profile did not achieve the most favorable readability profile, whereas the model with the best readability performance did not attain the highest overall quality scores. This pattern indicates that patient-facing AI systems require simultaneous optimization across multiple dimensions, including clinical completeness, factual accuracy, hallucination avoidance, safety-risk minimization, plain-language formulation, visible source support, actionable triage guidance, and appropriate expression of uncertainty.

The present default-prompt findings should therefore be regarded as a baseline against which constrained prompting and evidence-grounded generation strategies can be evaluated, rather than as evidence that the identified readability and transparency limitations are immutable characteristics of the evaluated models.

In the context of acute pancreatitis, LLM-generated patient education should incorporate concise explanations, explicit red-flag symptoms, clear thresholds for urgent medical attention, conditional framing for management decisions, avoidance of unsupported or unsafe treatment advice, and guidance to seek individualized clinical evaluation. Improvements in source attribution and currency signaling are also warranted. In the absence of these elements, responses may appear authoritative while remaining insufficiently understandable or transparent for patients making time-sensitive or uncertainty-laden decisions.

### Limitations

This study has several limitations. First, constraints in the question-development process relate to the public-source retrieval pathway. Because the original exploratory browsing logs were not retained as a formal archival dataset, the public-source retrieval pathway was retrospectively reconstructed. Accordingly, this component should be interpreted as a transparent operationalization of retained public search intents rather than a fully auditable prospective search log. Because the reconstruction was aligned to the finalized benchmark rather than derived from a contemporaneously archived search log, the public-source component should not be interpreted as evidence of unbiased prospective sampling from online query ecosystems. In addition, only a single broad seed term was used for each public platform, meaning that the resulting intents predominantly reflect mainstream information needs under general search conditions and may not capture long-tail, highly specific, or individualized query behaviors. To partially address this limitation, the final benchmark was balanced across prespecified clinical domains, source categories, and task types, and guideline-derived items were incorporated to ensure coverage of decision-critical acute pancreatitis content.

Second, the benchmark comprised 24 questions and 120 model responses and was deliberately confined to a single disease, acute pancreatitis. Although intentionally balanced across domains and source types, this sample represents a constrained subset of potential acute pancreatitis information needs. The study was designed to assess performance on a structured, decision-critical benchmark rather than to estimate model behavior across the full spectrum of real-world patient queries or other clinical conditions. Accordingly, the findings should be interpreted as a controlled public-interface snapshot of acute pancreatitis information rather than a comprehensive characterization of model performance across decision-critical diseases or broader clinical reasoning tasks. The observed relationships among informational quality, visible transparency-related features, and readability may differ in conditions with different levels of urgency, diagnostic uncertainty, treatment complexity, longitudinal self-management burden, or risk of harm.

Third, all prompts and model outputs were standardized in English, and readability was assessed using English-language formulas only. This approach strengthened methodological consistency for cross-model comparison and readability evaluation, but it limits generalizability to Chinese-language and multilingual patient education contexts. Because some source materials originated from Chinese-language public queries and guidelines, bilingual adaptation was required. Although efforts were made to preserve core clinical intent and minimize wording-driven variability, translation and standardization may have introduced changes in phrasing, cultural nuance, or perceived reading difficulty.

Fourth, the benchmark deliberately incorporated guideline-derived and decision-critical questions. Several topics, including ERCP timing, severity stratification, imaging strategies, antibiotic use, and nutritional management, are inherently complex even when translated into patient-facing language. Such prompts may therefore elicit more technical responses than organically generated layperson FAQs and may have contributed to the elevated reading-grade levels observed. Future studies should prospectively distinguish between layperson-derived queries and guideline-informed decision-critical prompts to assess whether readability limitations persist across both categories.

Fifth, only a single response was collected for each model-prompt pair in the primary analysis. This approach was selected to approximate the default patient-facing public-interface scenario, but it limits direct assessment of ranking stability across repeated generations. LLM outputs may vary across repeated generations, and public-interface models are subject to temporal drift due to updates in model weights, system instructions, safety layers, retrieval mechanisms, or interface configurations. Consequently, the primary analysis represents a single-time public-interface snapshot rather than a full assessment of within-model response stability across repeated sampling. To partially address this limitation, we added an exploratory repeated-generation sensitivity analysis using six representative prompts and two additional generations per model-prompt pair. This analysis suggested that the main quality-ranking pattern was broadly stable in the selected subset, but it could not eliminate uncertainty related to longer-term temporal drift, larger prompt sets, or future model updates. Therefore, rankings based on closely performing models should be interpreted cautiously. A further limitation relates to potential stylistic unblinding. Despite removal of model names, interface labels, logos, timestamps, and other identifiers prior to scoring, LLMs may still exhibit distinguishable stylistic signatures, including differences in formatting density, lexical patterns, and explanatory structure. To address this issue, we added an exploratory post-hoc model-identity guessing subset sensitivity analysis. Neither rater-specific nor pooled guessing accuracy was significantly higher than the 20% random probability, suggesting no consistent evidence that raters could reliably identify model sources from writing style alone in the selected subset. However, because the analysis was limited to a balanced 20-response subset, partial stylistic recognition cannot be fully excluded.

Sixth, although we added a structured response-level safety analysis for factual inaccuracies, clinical hallucinations, and clinical safety-risk severity, this assessment was not a fully exhaustive claim-by-claim verification of every biomedical statement. More importantly, the investigator-developed clinician-rated rubric represents a proxy for potential harm and was not designed or externally validated to predict patient comprehension, decision-making, or real-world behavior. The designation ‘low risk’ denotes a score of 1 under the predefined rubric and should not be interpreted as empirical evidence that an identified inaccuracy or hallucination would be harmless in real-world use. Patients were not exposed to the responses in the present study, and we did not assess comprehension, recall, confidence calibration, intended action, or actual health-related behavior. The analysis also did not account for health literacy, numeracy, previous disease experience, language proficiency, cultural context, or digital literacy. Expert clinicians may recognize and mentally correct minor inaccuracies that remain confusing or behaviorally consequential for patients, particularly in time-sensitive situations. Citation-level source verification was also limited because most unprompted responses did not provide conventional verifiable citations. Future studies should therefore combine granular claim extraction, citation-level validation, guideline-concordance mapping, and prospective patient-level comprehension and behavioral testing.

Seventh, the JAMA benchmark has inherent construct limitations when applied to conversational LLMs. Originally designed for static online health information, it was not intended for AI-generated dialogic outputs. As a result, authorship and disclosure components may underestimate platform-level transparency when such information is provided outside the response text. Nevertheless, response-level transparency remains clinically relevant, as patients commonly evaluate the content directly presented to them rather than auxiliary platform documentation.

Eighth, readability was assessed using formula-based metrics derived from a single readability calculator. Consistent use of the same tool across all responses enhanced internal comparability across models; however, minor variability may arise across different calculators due to implementation-specific differences in word, sentence, and syllable counting rules. Moreover, readability formulas primarily capture surface-level linguistic features, including sentence length, word length, syllable counts, and character patterns. They do not fully reflect patient comprehension, numeracy demands, actionability, information layout, emotional burden, cultural context, or the ability to correctly apply information in real-world clinical decision-making.

Ninth, inter-rater agreement varied across outcome measures. Inter-rater agreement was only moderate for EQIP percentage scores, despite excellent or almost perfect agreement for DISCERN, GQS, and JAMA benchmark scores. This may reflect the greater subjectivity and item-level interpretive complexity of EQIP scoring. Because raters were blinded to model identity, EQIP-related variability was more likely to introduce measurement noise, reduce precision, and attenuate or destabilize between-model differences than to systematically favor a particular model. Nevertheless, EQIP-based rankings and pairwise comparisons, particularly where effect sizes were small or confidence intervals were wide, should be interpreted more cautiously than outcomes supported by stronger inter-rater reliability.

### Future directions

Future research should assess LLM-generated patient-facing information under more realistic, clinically rigorous, and cross-disease conditions (30). Although the empirical findings of the present study should not be directly generalized beyond acute pancreatitis, the methodological framework may be transferable to other decision-critical diseases if disease-specific domains, guideline anchors, and safety rubrics are prospectively adapted. A practical next step would be to apply the same domain-balanced and source-balanced workflow to multiple high-risk conditions, such as acute coronary syndrome, stroke, sepsis, diabetic ketoacidosis, acute abdominal pain, and high-risk medication counseling. Across diseases, future studies should retain common evaluation dimensions, including informational quality, response-level transparency, readability, actionability, factual accuracy, hallucination frequency, guideline concordance, and safety-risk severity, while adapting prompt domains and clinical risk criteria to each condition. Cross-disease validation should stratify prompts by urgency, diagnostic uncertainty, treatment decision-making, discharge or self-management needs, and potential harm from delayed or incorrect action. Repeated generations should also be sampled to quantify within-model variability, ranking stability, and whether model performance patterns are consistent across diseases. For acute pancreatitis specifically, expanded prompt sets should be constructed from multiple public-query sources, authentic patient questions, clinician-identified safety-critical scenarios, and post-discharge self-management needs, with particular attention to deterioration recognition, imaging decisions, antibiotic use, ERCP indications, nutritional management, recurrence prevention, claim-level accuracy, citation verification, and guideline concordance. Future work should also combine response-level safety scoring with granular claim-level fact checking, citation-level validation, guideline-concordance mapping, and prospective patient-comprehension or behavioral-impact testing to determine whether observed safety signals translate into real-world misunderstanding or harmful action.

A feasible patient-validation study could adopt a two-stage design. First, a randomized, blinded, crossover experiment could compare original LLM responses containing clinician-adjudicated low-risk inaccuracies or hallucinations with clinician-corrected responses matched for length, structure, and readability. Adults with previous acute pancreatitis or caregivers involved in acute pancreatitis management could be stratified into low, intermediate, and adequate health-literacy groups using a validated instrument. Under standardized simulated scenarios, the primary outcome could be the frequency of behaviorally consequential decision errors, including delayed selection of emergency evaluation, inappropriate self-management, incorrect treatment expectations, and failure to recognize red-flag symptoms. Secondary outcomes could include objective comprehension, immediate and delayed recall, perceived clarity, trust, confidence, and confidence–accuracy calibration. Mixed-effects models could evaluate the interaction between response type and health-literacy level while accounting for repeated observations within participants and variation across scenarios. Because exposure to inaccurate information during active clinical care would be unethical, participants should receive immediate correction and standardized debriefing after each simulated task. A subsequent clinician-supervised pragmatic phase could examine whether comprehension performance predicts real-world information-seeking, unscheduled consultations, delayed presentation, inappropriate self-medication, and adherence to follow-up recommendations, while ensuring that all information used in actual care has been clinician approved.

Future work should determine whether the observed deficiencies are modifiable through constrained prompting, retrieval-augmented generation, model self-verification, or clinician-designed response templates (31, 32). A prospective multi-arm experiment could compare default responses with responses generated under: (1) a plain-language constraint, such as a sixth-grade reading level and short-sentence requirement; (2) mandatory citation of identifiable and current sources; (3) explicit red-flag and urgent-care reminder framing; (4) a combined prompt incorporating all three constraints; and (5) the combined prompt supported by retrieval-augmented generation using a locked, version-controlled corpus of current acute pancreatitis guidelines and clinician-approved patient-education materials. The same models and benchmark questions should be used across conditions, with repeated generations to quantify stochastic variability. Outcomes should include informational quality, readability, response-level transparency, claim-level factual accuracy, citation validity, guideline concordance, hallucination frequency, clinical safety-risk severity, patient comprehension, and simulated behavioral errors.

These interventions should be evaluated as multi-objective optimization strategies rather than according to improvement in a single score. Plain-language prompting may improve readability while omitting clinically necessary qualifications; mandatory citation instructions may increase visible transparency while generating fabricated or unverifiable references; red-flag framing may improve triage safety while increasing unnecessary alarm; and retrieval-augmented generation may improve evidence grounding while introducing outdated, conflicting, or contextually inappropriate retrieved content. A clinically acceptable strategy should therefore demonstrate improved readability and transparency together with preserved informational completeness, factual accuracy, and safety. For retrieval-augmented systems, the source corpus, document versions, retrieved passages, and response-level citations should be recorded and independently verified. The most promising configurations should subsequently undergo clinician review and health-literacy-stratified patient-comprehension and behavioral testing before direct patient-facing deployment.

A clinically appropriate patient-facing LLM response for acute pancreatitis should ideally be concise, plain-language, evidence-informed, and safety-oriented. It should clearly differentiate general educational content from urgent-care guidance, explicitly state red-flag symptoms, avoid overly rigid universal recommendations, and emphasize the need for individualized clinical evaluation. Evaluation of such outputs should extend beyond readability metrics to include patient comprehension testing, clinician-led safety review, and real-world usability assessment.

## Conclusion

In this benchmark of patient-facing acute pancreatitis information, publicly accessible LLMs produced responses with heterogeneous informational quality, limited visible transparency-related signals, consistently suboptimal readability, and a small number of clinician-adjudicated score 1 (low-risk) safety signals. Although certain models generated comparatively higher-quality and more comprehensive responses, no individual output satisfied all predefined readability targets, and higher quality scores did not necessarily establish factual accuracy, clinical safety, patient-education-level readability, or stronger response-level transparency. These findings indicate that current public-interface LLM outputs may function as preliminary drafts or adjunct tools for clinician-mediated patient education, rather than as standalone patient-facing materials. Future AI health-information systems should be optimized not only for informational completeness and perceived quality, but also for factual accuracy, hallucination avoidance, safety-risk minimization, readability, transparency, actionability, and clinically supervised safeguards.

## Data Availability

The original contributions presented in the study are included in the article/Supplementary material, further inquiries can be directed to the corresponding author/s.
